# Promotion of tumor progression and cancer stemness by *MUC15* in thyroid cancer via the GPCR/ERK and integrin-FAK signaling pathways

**DOI:** 10.1038/s41389-018-0094-y

**Published:** 2018-11-12

**Authors:** Cheolwon Choi, Nguyen Thi Thao Tran, Trinh Van Ngu, Sae Woong Park, Min Suk Song, Sung Hyun Kim, Yun-Ui Bae, Penchatr Diskul Na Ayudthaya, Javaria Munir, Eunbit Kim, Moo-Jun Baek, Sujung Song, Seongho Ryu, Kee-Hyun Nam

**Affiliations:** 1 Soonchunhyang Institute of Med-bioscience (SIMS), Soonchunhyang University, Cheonan, Korea; 2 Department of Microbiology & Immunology, Weill Cornell Medical College, New York, USA; 3 Department of Life Sciences, Yeungnam University, Gyeongsan, Korea; 4 Department of Physiology, Kyung Hee University, School of Medicine, Seoul, Korea; 5 Department of Surgery, College of Medicine, Soonchunhyang University, Chonan, Korea; 6 Department of Surgery, College of Medicine, Yonsei University, Seoul, Korea

## Abstract

Thyroid cancer is the fifth most common cancer diagnosed in women worldwide. Notwithstanding advancements in the prognosis and treatment of thyroid cancer, 10–20% of thyroid cancer patients develops chemotherapeutic resistance and experience relapse. According to previous reports and TCGA database, *MUC15* (*MUCIN* 15) upregulation is highly correlated with thyroid cancer progression. However, the role of *MUC15* in tumor progression and metastasis is unclear. This study aimed to investigate factors mediating cancer stemness in thyroid cancer. *MUC15* plays an important role in sphere formation, as an evident from the expression of stemness markers including *SOX2*, *KLF4*, *ALDH1A3*, and *IL6*. Furthermore, ectopic expression of *MUC15* activated extracellular signal-regulated kinase (*ERK*) signaling via G-protein–coupled receptor (GPCR)/cyclic AMP (cAMP) and integrin/focal adhesion kinase pathways. Interestingly, ectopic expression of MUC15 did not affect *RAF*/mitogen-activated protein kinase kinase (*MEK*)-mediated *ERK* activation. The present findings may provide novel insights into the development of diagnostic, prognostic, and therapeutic applications of *MUC15* in thyroid cancer.

## Introduction

According to reports of National Cancer Institutes (NCI), thyroid cancer has shown a significant increase over the last 30 years^1^. Although thyroid cancer has a good prognosis and is considered easily curable via surgical resection and radio-iodine based therapies, 10–20% of thyroid cancer cases involve aggressive behavior including local invasion, distant metastasis, drug resistance, recurrence, and mortality^2–5^. Since thyroid carcinoma is at a high risk of invasion, recurrence, and metastasis, it is important to study the underlying molecular mechanism.

A crucial cancer stem cell (CSC) population causes these malignant phenotypes in various cancers^6–10^, especially thyroid cancer^11,12^. Indeed CSCs can be verified through clonogenic assays of cells to assess proliferative capacity including sphere formation in vitro^13,14^ or via in vivo tumorigenesis experiments^15^. Current radiotherapy and chemotherapy often eliminate the bulk of cancer cells but not CSCs, which are protected via specific resistance mechanisms^16,17^. These unsolved issues may be explained on the basis of CSC-like properties of many tumor types^6,11,18,19^. We found that one isoform of mucins, *MUC15* play a critical role of mediating cancer stemness in thyroid cancers.

Mucins are high-molecular-weight membrane glycoproteins (>200 kDa) in various types of epithelial cells^20–22^. Secreted form of mucins have a protective functional epithelial barrier to protect against from bacteria and virus infections^21^ while membrane-associated-mucin proteins are intracellular receptors involved in signal transduction, leading to coordinated cellular responses including proliferation, differentiation, apoptosis, and secretion of specialized cellular products^23,24^.

*MUC15* upregulation is significantly correlated with various types of cancers such as colon cancer, hepatocellular carcinoma, and especially thyroid cancer^25–27^. High cancer scores of MUC15 expression are significantly correlated with age, distant metastasis, and the presence of multifocality^26^. In addition, ectopic *MUC1* expression upregulates CSC markers in breast cancer and lung cancer such as *OCT4*, *SOX2*, *ALDH1A1*, *IL6*, and *CXCR4*^17,28,29^. Moreover, *MUC1* upregulation has been considered to significantly contribute to the aggressiveness of the papillary thyroid cancer (PTC)^30^. Similarly, *MUC4* is known to be upregulated in 20% of PTC cells compared to normal cells^26,31^ and is correlated with the development of chemoresistance in pancreatic CSC^32^. These results imply that mucins play an important role in mediating tumor development and progression and are related to cancer stemness properties in thyroid cancer and various types of cancer^17,29,33,34^. However, its physiological role and its underlying molecular mechanisms in thyroid cancer progression and metastasis are unclear.

The *RAS*-*ERK* pathway mediates cancer progression, metastasis, and cancer stemness in various types of cancer^35–37^. Furthermore, G-protein-coupled receptor (GPCR) and integrin-focal adhesion kinase (*FAK*) signaling are upstream targets of activated extracellular signal-regulated kinase (*ERK*)^38,39^, thereby increasing the stemness of thyroid cancer cells.

This study aimed to investigate the correlation between CSC-like properties and thyroid cancer progression and metastasis because CSCs are crucial for cancer recurrence, metastasis, and drug resistance, which are yet unclear. Our results may provide novel insights into help understand physiological and cellular mechanisms of recurrence and metastasis in thyroid cancer and the characteristics of thyroid CSC, and in developing novel therapeutic targets for thyroid cancer patients.

## Results

### MUC15 is upregulated in later stages of thyroid cancer

**Fig. 1:**
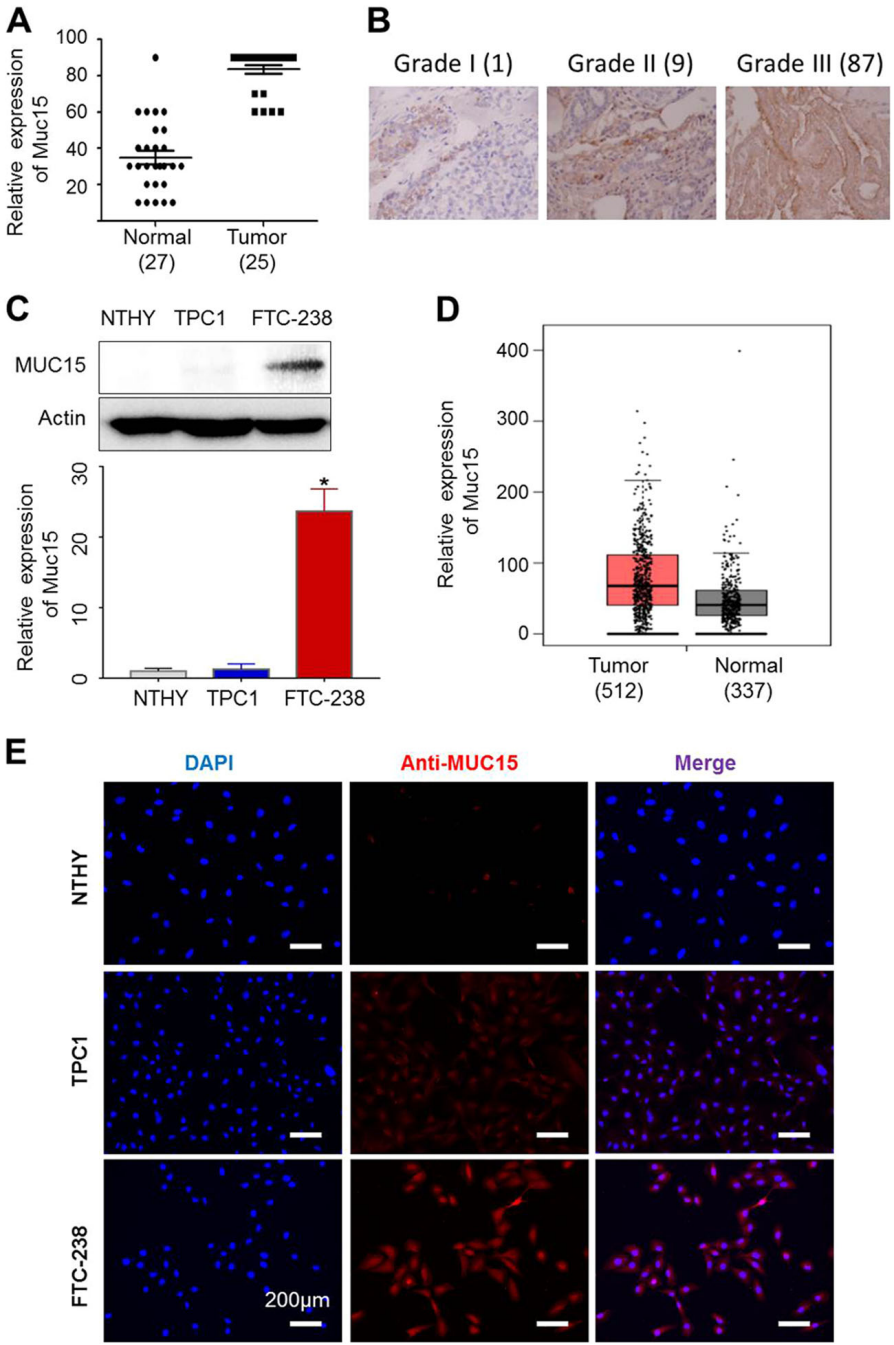
MUC15 is highly expressed in Thyroid cancer tissue and cell line. **a** Relative expression level of *MUC15* between tumor (25 cases) and their neighboring normal thyroid tissue (27 cases) in thyroid cancer patients. **b** *MUC15* Immunohistochemical (IHC) positivity in the patients was scored from 0 to 3 according to the percentage positivity (grade 0, 0%; grade 1, 1–33%; grade 2, 34–66%; grade 3, 67–100%). **c** Relative expression level of *MUC15* normalized by *GAPDH* expression level among Nthy-ori-3-1, TPC-1, and FTC-238 cells. Western blots showing the expression level of the *MUC15* protein. **d** Thyroid tumors have enhanced expression of MUC15 in patients according to TCGA database. **e** Immunostaining of *MUC15* in Nthy-ori-3-1, TPC1 and FTC-238 cells, respectively

Thyroid tumors display greater *MUC15* expression than neighboring normal thyroid epithelial cells^26^. These findings are concurrent with those of our clinical studies. Mostly thyroid tumors in patients have greater *MUC15* expression than normal tissue, especially in higher-grade tumors (Fig. 1a, b). To clarify the role of *MUC15* in thyroid cancer progression, we first examined *MUC15* expression in thyroid cancer cells (FTC-238 and TPC-1), relative to that of normal thyroid cells (Nthy-ori-3-1). Metastatic thyroid cancer cells (FTC-238) displayed upregulated *MUC15* expression compared to normal thyroid epithelial cells; in particular, FTC-238 cells displayed significantly upregulated *MUC15* expression (greater than 20 folds) than Nthy-ori-3.1 cells (*p* = 0.0014); however, TPC-1 (non-metastatic cancer cells) did not display a significant difference (Fig. 1c and Supplementary Figure S1D). Consistent with reverse transcription PCR (RT-PCR) results, western blot as well as and immuno-fluorescence analyses confirmed increased *MUC15* expression in thyroid cancer cells (Fig. 1e). These findings are confirmed via TCGA database using GEPIA website based on a published dataset at 2014 (http://gepia.cancer-pku.cn/) (Fig. 1d and Supplementary Figure S1A)^40,41^. In addition, thyroid cancer patient with high level of *MUC15* show worse prognosis analyzed by cBioPortal (http://www.cbioportal.org/) and GEPIA (http://gepia.cancer-pku.cn/) websites respectively (Supplementary Figure S1B, C)^41,42^.

### Ectopic MUC15 expression promotes CSC-like properties

**Fig. 2:**
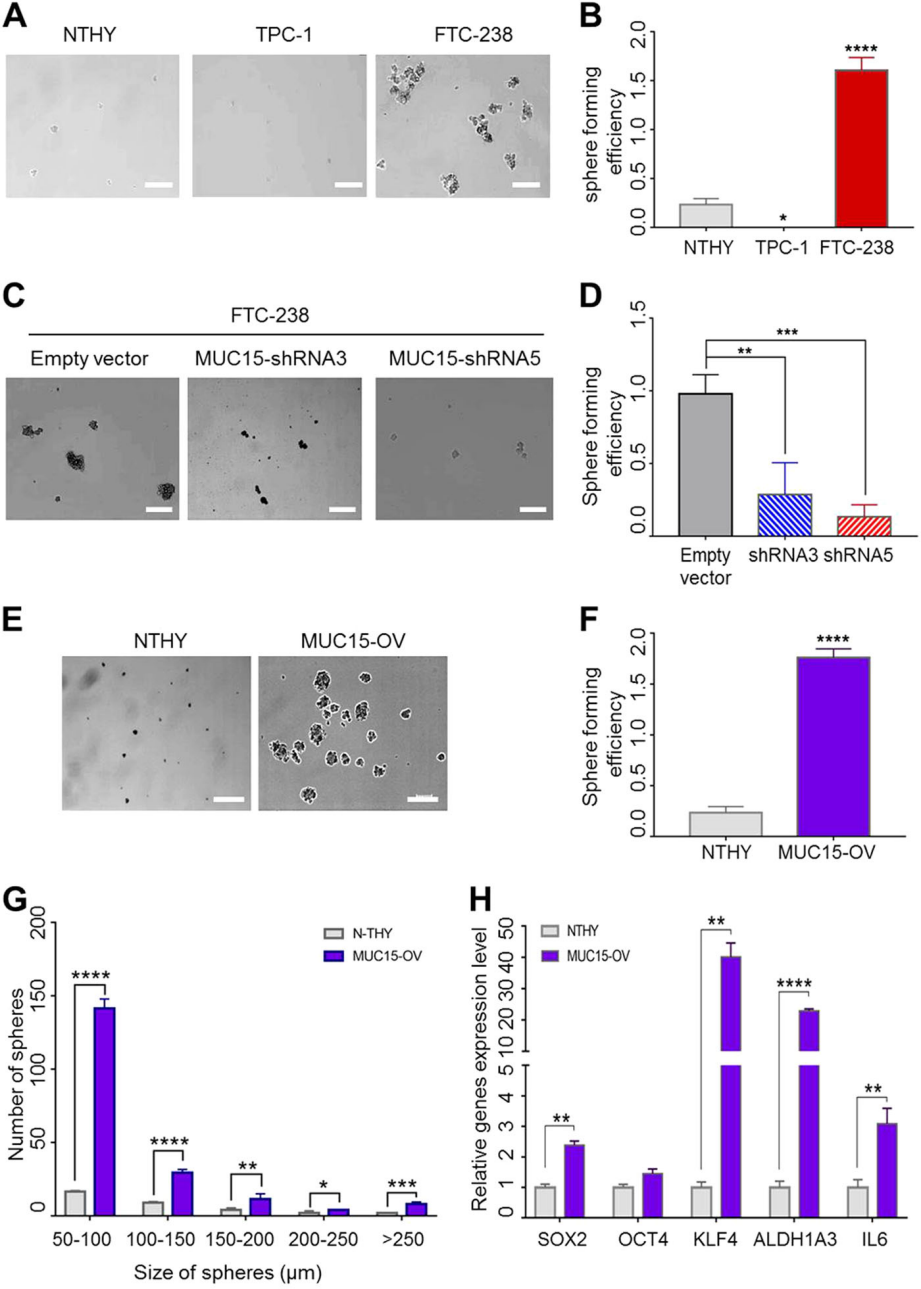
Over-expression of *MUC15* drives cancer stem cell properties. **a**, **b** Representative images showing sphere formation among Nthy-ori-3-1 cells, TPC-1 and FTC-238 cells and quantitative values measured size of sphere from the results. **c**, **d** Representative images show sphere formation ability among transfected with Empty vector and indicated *MUC15* shRNA respectively and quantitative values measured size of sphere from the results. **e**–**g** Representative images showing sphere formation between Nthy-ori-3-1 and *MUC15*-OV (*MUC15* overexpression) cells and quantitative values measured size of sphere from the results. **h** RT-qPCR data show that *MUC15* over expression cell increased the expression of stem cell markers *SOX2*, *OCT4*, *KLF4* and cancer stem cell markers *ALDH1A3* and *IL6*

Metastasis and chemotherapeutic resistance are associated with properties of CSCs in various types of cancers^18,43–45^. Sphere formation is a key characteristic of stem cells and CSCs; hence, this characteristic is harnessed in analyzing properties of cancer stemness^34,46–48^. While investigating the physiological role of *MUC15* in thyroid cancer, we found that FTC-238 cancer cells display greater sphere formation abilities; however, Nthy-ori-3-1 and TPC-1 cells did not adequately generate spheres (Fig. 2a, b).

To confirm the function of *MUC15* in sphere formation, we knocked down *MUC15* in FTC-238 with lentiviral *MUC15* shRNA (Supplementary Figure S2A and C). Consequently, sphere formation abilities of *MUC15* shRNA-transduced cells were lower than those of control cells (cells transfected with the vector control) (Fig. 2c, d). These data together suggest *MUC15* expression is strongly associated with sphere formation among thyroid cancer cells.

To further verify whether *MUC15* regulates sphere formation, we transduced *MUC15* expressing lentivirus to Nthy-ori-3-1 cells to generate cells stably overexpressing *MUC15*, referred to as *MUC15* overexpress cells (*MUC15*-OV cell) (Supplementary Figure S2B, D, E and F). Interestingly, these cells displayed enhanced sphere formation ability, implying CSC-like properties (Fig. 2e, f). We also quantified the number and size of spheres between Nthy-ori-3-1 and *MUC15*-OV cells. Although both Nthy-ori-3-1 and *MUC15*-OV cells could form spheres of 50–100 and 100–150 µm, *MUC15*-OV cells exhibited numerous colonies. In particular, spheres of greater than 250 µm were only found in *MUC15*-OV cells (Fig. 2g).

The expression level of stem cell markers *SOX2*, *OCT4*, and *KLF4* in *MUC15*-OV cells were 2.38, 1.45, and 40.1 folds higher than those in normal thyroid epithelial cells, NThy-ori-3.1, respectively (Fig. 2h). Additionally, the expression level of *ALDH1A3*, a CSC marker among various types of cancer cells^49^, and *IL6* known as a downstream functional target to induce sphere formation in many types of cancer cells^50–53^, were 22.9 and 3.09 folds higher in *MUC15*-OV cells than in Nthy-ori-3.1 cells (Fig. 2h).

In addition, ectopic *MUC15* expression increases cellular survival and proliferation even in adhered cells. The data show that the number of *MUC15*-OV cells was higher than that of Nthy-ori-3.1 cells during suspension culture (Supplementary Figure S3A). Moreover, *MUC15*-OV cells showed a low population of apoptotic cells in suspension culture, especially 3 and 5d after incubation, as determined via FACS (Supplementary Figure S3B, C). Since *MUC15* could inhibit apoptosis, thereby increasing the number of survived cells in suspension culture, this property is correlated with metastasis and CSC-like properties^25^.

### MUC15 activates the MEK-ERK pathway independent of BRAF

**Fig. 3:**
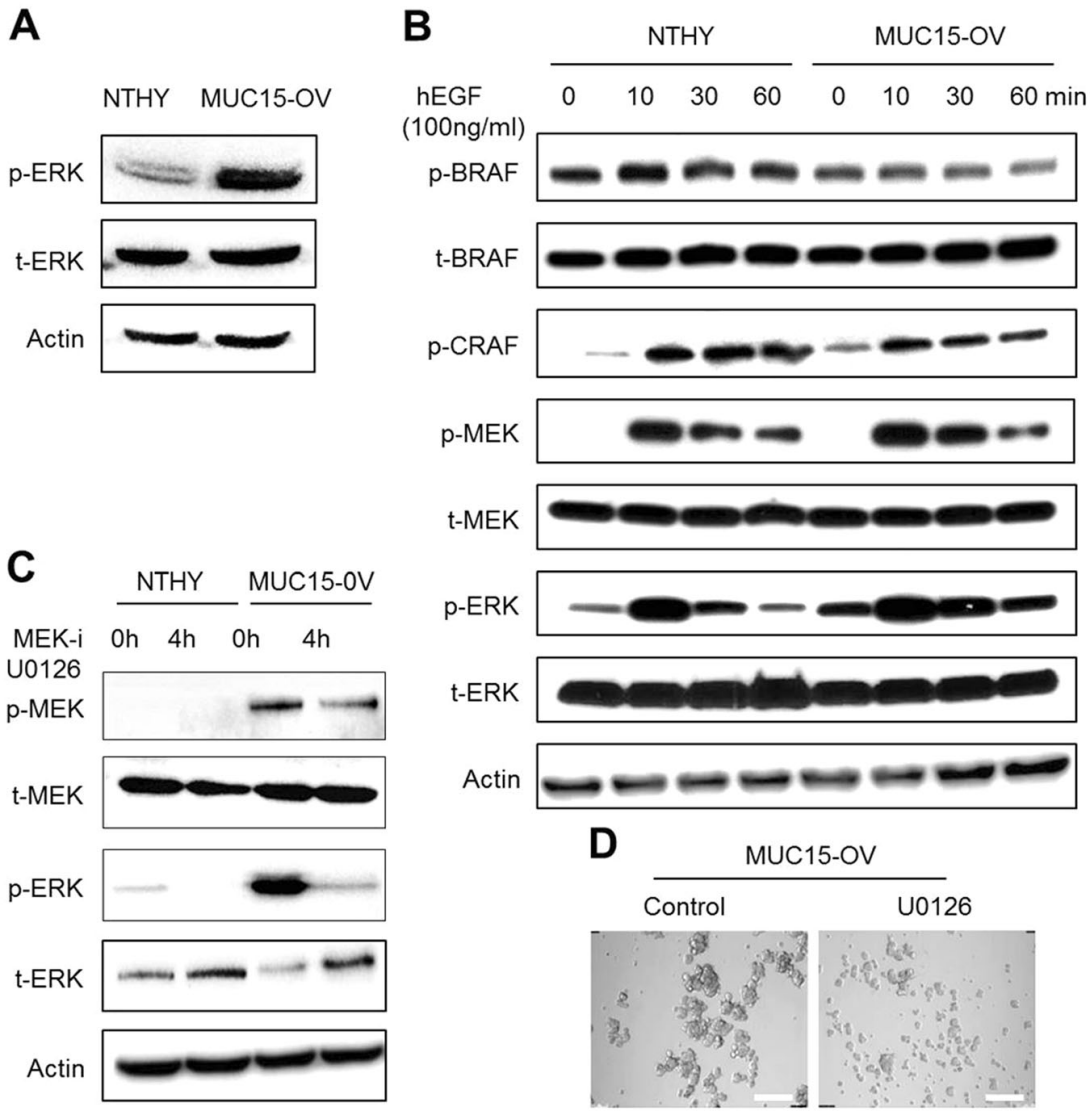
MUC15 drives activation of *MEK*/*ERK* independent to *BRAF*. **a** The level of phosphorylation ERK1/2 and total ERK1/2 in *MUC15* over expression cell are higher than Nthy-ori cell. **b** Nthy-ori and *MUC15* over expression cells were treated with either serum or hEGF for 10, 30, 60 min and RAF/MEK/ERK protein expression were determined by immunoblotting. **c**, **d** Blocking ERK activity by UO126 (10 µM) were determined by immunoblotting and inhibition of ERK decreased mammosphere formation ability of *MUC15* over expression cells

*MUC15* induces the activation of oncogenic *ERK* activation, thereby mediating metastasis in colon cancer^25^. *MUC1* could activate ERK-C/EBP beta signaling in breast cancer cells^28^. *MUC4* triggers *ERK* activation dependent on confluence of mammary epithelial and tumor cells^54^. These findings together suggest that *MUC15* may also activate *ERK* signaling in thyroid epithelial cells. Thus, we investigated whether over-expression of *MUC15* increases ERK phosphorylation. Indeed, *MUC15*-OV cells showed increased MEK and ERK phosphorylation (Fig. 3a). However, we could not observe differences in BRAF activation on adding hEGF (100 ng/ml) between Nthy-ori-3.1 and *MUC15*-OV cells, although the initial state of MEK and ERK phosphorylation was enhanced in *MUC15*-OV cells (Fig. 3b). Previously, activation of *ERK* is associated with CSC-like properties and sphere formation^34^. ERK suppression with pharmacological inhibitor UO126 decreased sphere formation in *MUC15*-OV cells (Fig. 3c, d). These observations suggest that *MUC15* plays an important role in developing CSCs via the *MEK*-*ERK* pathway.

### MUC15 induces expression of genes related to the GPCR-cAMP pathway, thereby increasing ERK phosphorylation

**Fig. 4:**
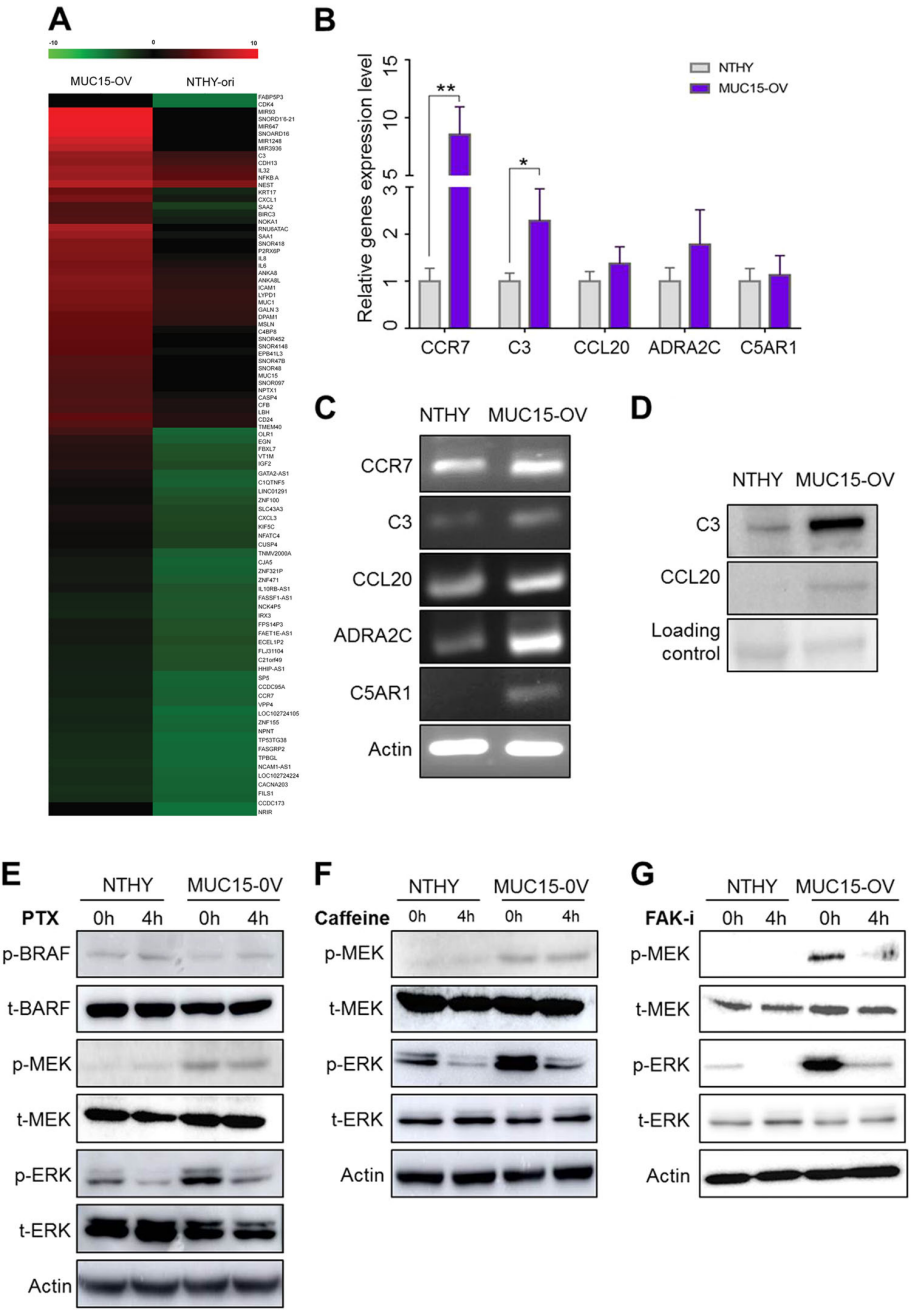
GPCR signaling pathway is candidate downstream target of *MUC15*. *MUC15* drives activation of *ERK* via *GPCR*-*cAMP* signaling pathway independent to *RAF*/*MEK*, associated to *FAK* signaling. **a** *GPCR* signaling was determined as a downstream target of *MUC15* by using RNA-SEQ analysis. **b**–**d** RT-PCR and RT-qPCR and western blot data represented the gene upregulated of *GPCR* signaling pathway in *MUC15* overexpression cell and Nthy-ori cell. **e**, **f** Nthy-ori and *MUC15* overexpression cell were treated with PTX, an inhibitor of *GPCR* and Caffeine, an inhibitor of *cAMP*. The protein expression was determined by immunoblotting. **g** Nthy-ori and *MUC15* over expression cells were treated PF-573228 as 10 μM, as an inhibitor of *FAK*, and protein levels of *MEK/ERK* were determined by immunoblotting

Sphere formation is regulated by various signaling pathways that promote CSCs. To determine signaling pathway involves *MUC15*, we performed next-generation sequencing (NGS) for total mRNA and compared the expression of numerous genes between Nthy-ori-3-1 and *MUC15*-OV cells, using a log_2_ scale heat-map via MeV program (Fig. 4a)^55^. Differentially expressed target genes were analyzed in silico to further analyze enriched signaling pathways through the Carcinogenic Potency Database (CPDB) molgen website (http://cpdb.molgen.mpg.de/) and GO-term analysis using PANTHER (http://pantherdb.org/)^56,57^. These two different tools revealed that the molecular signaling pathway related to *MUC15* is the *GPCR* pathway (Supplementary Figure S4A, B, Table S1 and Table S2). We confirmed the expression of *GPCR*-related genes in response to *MUC15* via RT-qPCR and western blot analysis (Fig. 4b–d). As shown in Fig. 4b; *CCR7*, *C3* expression level in *MUC15*-OV cell was greater than eight-folds and two-folds than those in Nthy-ori-3.1 cells, respectively.

Together, RNA-SEQ analysis and western blot analysis displaying that over-expression of *MUC15* mediates activation of *ERK*, we hypothesized that *MUC15* may induce activation of *ERK* via a *GPCR* pathway rather than *RAS*-*RAF* pathway, in accordance with a previous study^58^. Previously, cells were treated with pertusis toxin (PTX) (200 ng/ml) to inhibit the *GPCR* signaling pathway^59^. PTX is known to catalyze the ADP-ribosylation of the αi subunits of the heterotrimeric G protein^60^. Interestingly, phosphorylation level of ERK of *MUC15*-OV cells was decreased with PTX treatment after 4 h (Fig. 4e). This inhibitor can block the activation of *ERK*, although phosphorylation levels of BRAF and MEK were not altered mediated by *MUC15* over-expression (Fig. 4e).

These results demonstrate that *MUC15* activate *GPCR*-*ERK* signaling; however, the involvement of other pathways cannot be eliminated. We were curious about which downstream signaling pathway mediates *GPCR*-*ERK* signaling independent on *MEK* activation. cAMP is known as the second messenger to activate *ERK* in endocrine cells mediated by *GPCR* signaling pathway independent of *MEK* activation^61^. Therefore, we wanted to investigate whether inhibition of cAMP production by caffeine treatment (1 mM) decreases the activation of *ERK* without affecting MEK phsophorylation^62^. Surprisingly, treatment with caffeine drastically decreased the activation of ERK, but not MEK in both Nthy-ori-3-1 and *MUC15*-OV cell lines (Fig. 4f).

These findings raise the question why *MUC15* may drive *ERK* activation via *GPCR*/CAMP pathway but stimulate *MEK*/*ERK* activation through another pathway. GPCR crosstalk with *FAK* signaling is critical to activate *ERK* activation^63^. MUC5AC mediates metastasis of cancer cells by interacting with integrin β4–FAK signaling in lung cancer cells^64^. We hence set to investigate whether *FAK* signaling may be associated with *ERK* activation triggered by *MUC15* over-expression. Interestingly, FAK inhibitor (PF-00562271) can suppress activation of *MEK* and *ERK* in *MUC15*-OV cells (Fig. 4g). Previous findings and these results together indicate that over-expression of *MUC15* could activate *MEK*-*ERK* signaling by the integrin-*FAK* signaling pathway.

### The GPCR-cAMP pathway is critical to sustain sphere formation and motile behavior of thyroid cancer mediated by MUC15 over-expression

**Fig. 5:**
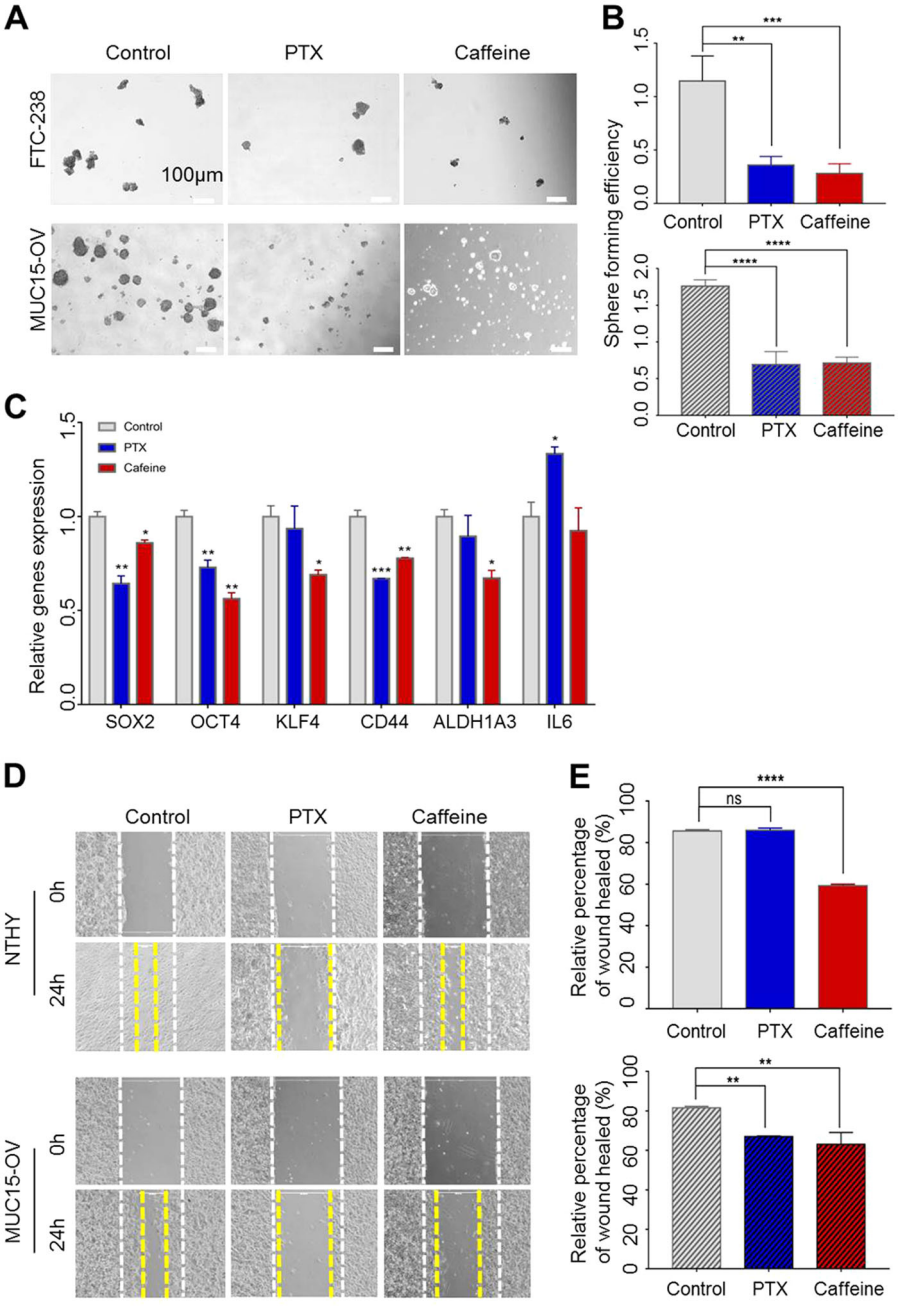
*GPCR*-*cAMP* pathway regulates motility and migration of thyroid cancer cells. **a**, **b** Sphere formation ability of cell lines express *MUC15*, *MUC15* overexpression cell and FTC-238, are reduced after treated with PTX (200 ng/ml) and Caffeine (1 mM). **c** The expression level of stem cell and cancer stem cell in *MUC15* overexpression cell were determined by RT-qPCR. **d**, **e** The migration of Nthy-ori cell and *MUC15* overexpression cell after treated with PTX (200 ng/ml) and caffeine (1 mM)

*MUC15* enhanced sphere formation and the *GPCR*-cAMP axis is important for the activation of *ERK* signaling mediated by *MUC15* (Fig. 4). Therefore, we investigated whether the *GPCR*-cAMP axis is involved in sphere formation in thyroid cancer. Interestingly, PTX or caffeine treatment reduces sphere formation ability of *MUC15*-OV cells and FTC-238 cells respectively (Fig. 5a, b). In addition, these drugs also decrease known stemness markers such as *SOX2*, *OCT4*, *KLF4 CD44*, and *ALDH1A3* (Fig. 5c). Together, we concluded that exogenously expressed *MUC15* induces the expression of genes related to the *GPCR* signaling pathway and triggers activation of the *GPCR*-cAMP-*ERK* pathway independent of the *RAF*/*MEK* signaling pathway.

However, the functional role of *MUC15* on cell migration is controversial. In hepatocellular carcinoma and trophoblast-like cells, *MUC15* decrease migration capability^27,65^. Whereas, in case of colon cancer, *MUC15* drives invasive migration through boyden chambers, thereby enhancing metastasis^25^. In our study, there was no major difference between Nthy-ori-3-1 and *MUC15*-OV cells for wound healing migration capability (Fig. 5d). However, inhibition of *GPCR* or cAMP signaling by PTX and caffeine, respectively, decreased wound healing migration in *MUC15*-OV cells (Fig. 5d, e). However, caffeine treatment selectively suppresses migration ability of *MUC15*-OV cells, but not Nthy-ori-3-1 cells (Fig. 5d, e). These observations imply that *MUC15* somehow has an inhibitory role of migration such as strong adhesion; however, activation of *GPCR*-cAMP signaling pathway compensates the ability of migration.

### MUC15 enhanced tumorigenesis in NOD/SCID mice

**Fig. 6:**
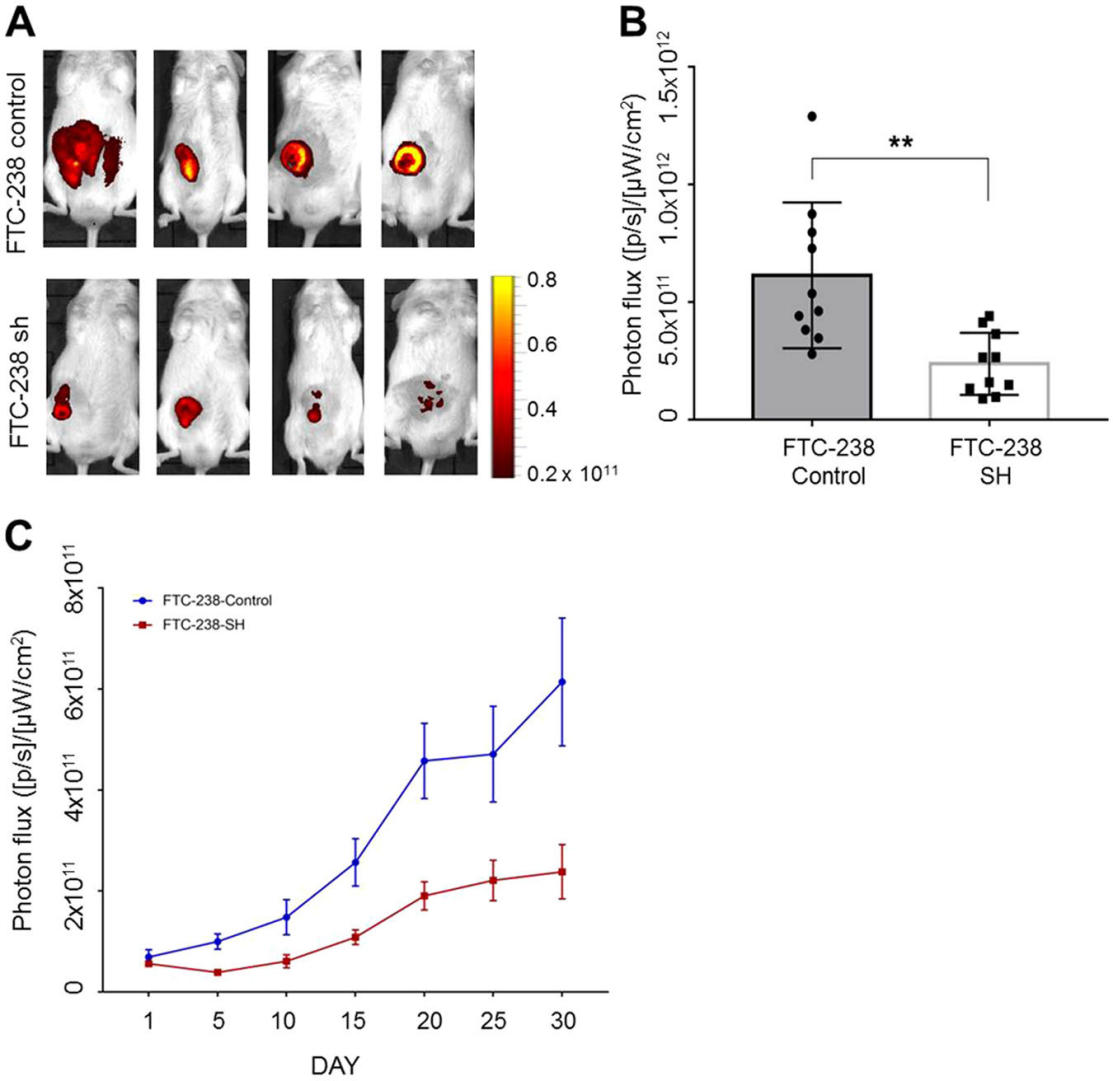
*MUC15* expression enhanced tumorigenesis in NOD/SCID mice. **a**, **b** The GFP signal imaging after 30 days inject FTC-238 control, FTC-238 *MUC15* knockdown (FTC-238-SH) respectively. **c** The radiant efficiency of GFP signals from 1 day to 30 day after injection thyroid cancer cells

To confirm cancer stem-like capacity driven by *MUC15* in vivo, we additionally performed a tumorigenesis experiment using a xenograft mouse model. First, we generated lentiviral-mediated control or *MUC15* shRNA knockdown cells in FTC-238, wherein *MUC15* was overexpressed, compared to Nthy-ori-3-1 and TPC-1 cells. These lentiviral vectors containing tGFP thereby transfected cells, which could then be detected by the IVIS imaging system. These cells were injected subcutaneously into the belly in NOD/SCID mice and the mice were monitored for up to 30 days. Interestingly, the signal of tGFP from FTC-238 control cell has significantly higher intensity than that of *MUC15* knockdown cells in all of monitoring (Fig. 6a–c and Supplementary Figure S5B). In addition, FTC-238 cells bearing control shRNA generated larger-sized tumors than those transfected with *MUC15* shRNA (Supplementary Figure S5A). These data indicate that *MUC15* is closely associated with tumorigenesis and carcinogenesis.

## Discussion

Regardless of cell type to be initiated, various types of tumors develop in the thyroid gland, such as papillary (the most common), follicular, medullary, and anaplastic (the least common) tumors. Although most types of thyroid tumors are benign, there are still some types of tumors that can be malignant and metastatic, recurrent, and even display chemotherapeutic resistance. Several studies confirmed various vital signaling pathways that modulate embryonic development and stem cell maintenance. However, aberrant regulation of these intrinsic biological processes may have harmful effects on neoplastic transformation. Nowadays, it is reported that normal stem cells can transform to CSC, thereby promoting malignancy^66^.

Cancer stemness is considered a critical feature to mediate malignancy of cancer such as metastasis, recurrence, drug resistance in many types of cancer as well as thyroid cancer^11,16^. It led us to investigate what factors mediate cancer stemness in thyroid cancer. We identified *MUC15* plays a key role in the progression of tumorgenesis by enhancing cancer stemness in thyroid cancer. Either gain or loss of function study demonstrates that *MUC15* is necessary and sufficient factor to induce feature of thyroid CSC. The *MUC15* expression is higher in follicular thyroid cancer cells, especially FTC-238 cell line, while TPC-1 cells derived from primary thyroid cancer does not show enhanced expression of *MUC15*. In consistent with previous studies, our results show that *MUC15* play a positive role of developing thyroid cancer progression^67^.

Our data indicate that *MUC15* expression enhanced sphere formation, the renewal characteristic of stem-like properties. In addition, *MUC15* expression increases several vital genes to mediate CSC properties, *SOX2*, *KLF4* and *IL-6* in normal thyroid epithelial cells (Fig. 2h)^51,53^. Consistent with the previous reports, *MUC15* indeed drives the invasive behavior of colon cancer and metastasis^25^. Collectively, our results suggest that *MUC15* play an important role in developing CSC thereby mediate metastasis and recurrence of thyroid cancer.

CSC are developed by hijacking intrinsic signaling pathway which mediates progression of normal embryonic development in stem cells. This pathway is also known as key player to modulate normal embryonic development as well^68,69^. The *GPCR* pathway is thought to be related to the stem cell function by the fact that the pattern of expression of *GPCR* varies greatly at each step during embryonic development or differentiation^70^. This ability allows tumor to be regenerated after anti-cancer therapy by chemotherapy or radiotherapy.

*GPCR* signaling pathway closely associated with cancer stemness in thyroid cancer according to previous studies^71^. Consistent with this, we found *MUC15* induces expression of several class of *GPCR* signaling related genes and confer properties of cancer stemness by the *GPCR* pathway. These genes that mediate *GPCR* signaling pathway are highly up-regulated in *MUC15*-OV cells such as *CCR7*, *C3*, *CCL20*, and *C5AR1* (Fig. 4b–d). *CCR7*, one of chemokine receptor involved in *GPCR* families, it is identified as tumor progression marker in thyroid cancer patients^72^. In the previous report, *CCR7* mediates activation of *ERK* thereby suppresses apoptosis in lung cancer^73^. *CCR7* also promotes mammary tumorigenesis through amplication of stem-like cell^74^. *CCL20* is identified as inflammatory factor to recruit dendritic cells^75^. Production of *CCL20* by lung cancer could induce cell migration and proliferation via *PI3K* signaling pathway as well^76^. Recent study suggests that inflammatory *CCL20* is also required to maintain CSC in breast cancer^77^. In addition, we found some up-regulated genes, *ADRA2C*, *C3*, and *C5AR1*, which were not previously reported in cancer stemness. These findings may provide novel candidate markers to identify CSC in thyroid as well as other types of cancer.

Important downstream target of *GPCR* signaling pathway is *cAMP*-*PKA* pathway. Subunit of *GPCR*, the Gαs and Gαi/o are able to trigger activation of adenylate cyclase to modulate production of cyclic AMP (cAMP) and cascadic activation of protein kinase A (PKA)^78,79^. Treatment of caffeine as inhibitor of adenylate cyclase suppresses the proliferation and migration of the cell expressing high level of *MUC15* (Fig. 5). This result indicates that cAMP plays a critical role to promote sphere formation and *ERK* activation mediated by *MUC15*. It is reported that caffeine consuming such as coffee uptake reduce the risk of several types cancers including breast, colon^80^. These clinical studies may associate with our finding that caffeine effectively decreases CSC properties and migration ability (Fig. 5).

**Fig. 7:**
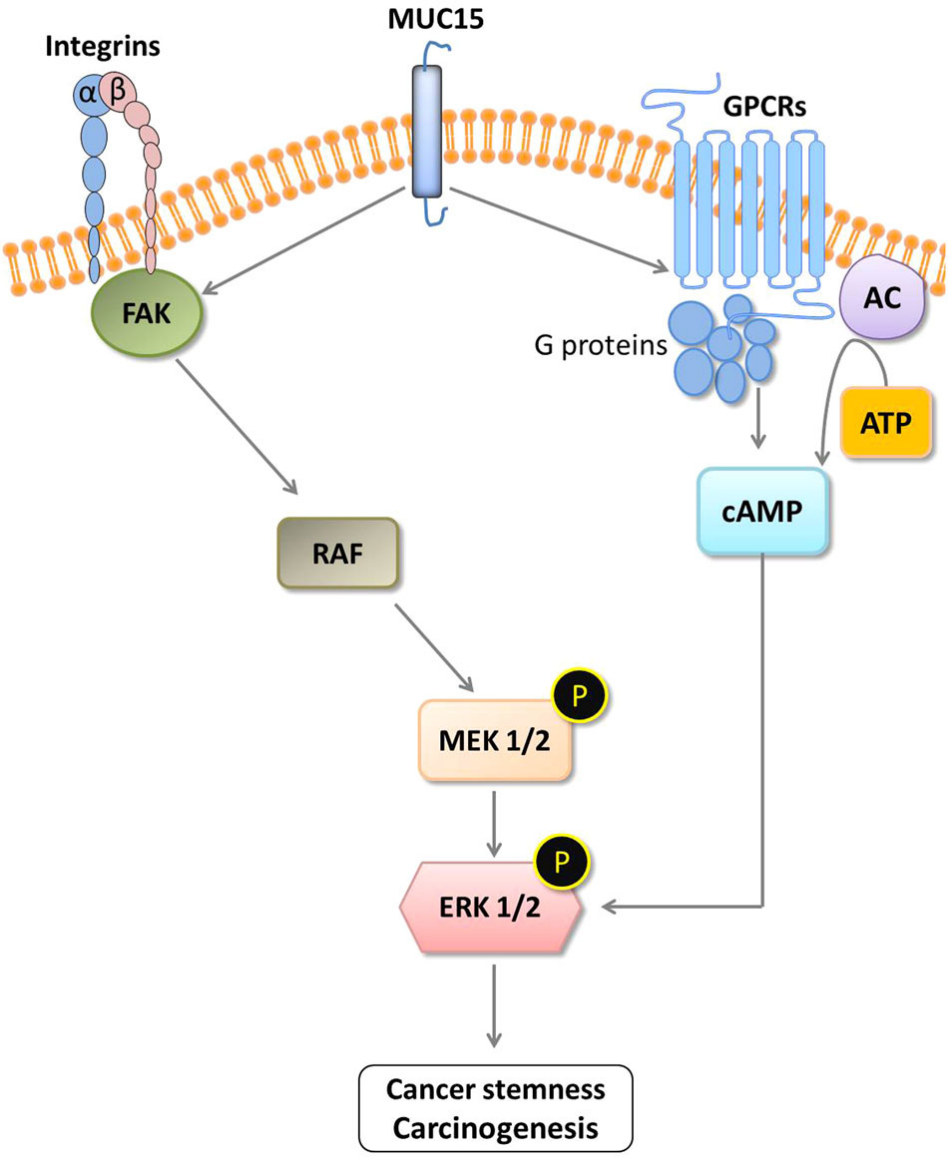
Schematic diagram of cell signaling pathway regulated by MUC15. Over-expression of MUC15 activate MEK-ERK pathway dependent on integrin-FAK and GPCR-cAMP pathway thereby promote cancer stemness and metastasis. Interestingly, cAMP signaling affects ERK activation independent on Raf-MEK signaling pathway driven by MUC15 over-expression

Ectopic expression of *MUC15* increase activation of *ERK* signaling pathway but *BRAF* does not seem to be a downstream target of *MUC15*, although *BRAF* reported in many case of thyroid cancer^81^. Furthermore, there are cross-talks between *GPCR* and integrin signaling in cell proliferation the activation of some *GPCR* component can activate *FAK* and stimulate the activation of *MEK*/*ERK* signal^82,83^, it is consistent with the activation of *FAK* and *GPCR* association with *MUC15* expression (Fig. 4e–g). Taken together, we suggest that *MUC15* mediated *GPCR*-*cAMP* signaling pathway, which crosstalk with integrin-*FAK* signaling are critical to driving sphere formation via *ERK* activation independent to *RAS*-*RAF* signaling (Fig. 7).

*MUC15*-mediated signaling pathway contributes a key characteristic of CSC and confers opportunities to development novel therapeutic strategies and diagnostic/prognostic markers for thyroid cancer patients. Further investigation of *MUC15*-mediated gene expression and downstream signaling pathway will elucidate self-renewal properties of CSC for highly tumorigenic population of CSC in thyroid cancer.

## Materials and methods

### Cell culture

The cell lines used to recapitulate human thyroid cancer were FTC-238, derived from follicular thyroid carcinoma (FTC) lung metastasis from a 42-year-old man, and TPC-1, derived from a weak metastatic papillary thyroid carcinoma (PTC)^70^. Human thyroid epithelial cell line Nthy-ori 3-1 constituted the control. Cancer cells were cultured with Dulbecco’s modified Eagle’s medium (DMEM) (Mediatech Cellgro, USA) containing 10% fetal bovine serum (FBS) (Gibco, USA), 100 IU/ml penicillin, and 100 µg/ml streptomycin (Invitrogen, USA). Epithelial cells were cultured with Roswell Park Memorial Institute medium (RPMI) (Mediatech Cellgro) containing 10% FBS 100 IU/ml penicillin, and 100 µg/ml streptomycin. Cells were cultured at 37 °C and 5% CO_2_.

### In vivo tumorigenicity assay

All animal work was conducted in accordance with a protocol approved by the Institutional Animal Care and Use Committee at Soonchunhyang University. *MUC15* tumorigenicity in vivo was investigated using a xenograft tumor model in the NOD/SCID mice. Eight-week-old male NOD/SCID mice were provided sterilized food and water and equally divided into three groups. Approximately 1 × 10^6^ cells with or without *MUC15* shRNA and control shRNA (vector control) were mixed with Matrigel (Corning, USA) and subcutaneously inoculated into the right flank of each NOD/SCID mice. These cells contain GFP-expressing vectors to monitor the GFP fluorescence signal using IVIS image system (Xenogen) every 5 days up to day 30. Once bearing palpable tumors (about 4 weeks after tumor cell inoculation), mice were euthanized and their tumors were isolated, and photographed. Experiments were performed in triplicate.

### Human samples

Human tumor samples were collected from patients enrolled on Institutional Review Board approved trials at Yonsei Hospital (IRB 4-2012-0682). Specimens were collected after obtaining written informed consent prior to undergoing any study-specific procedures in accordance with the Declaration of Helsinki. Patient’s identity of pathological specimens remained anonymous in the context of this study.

### RNA extraction and cDNA synthesis

In order to perform RT-qPCR and NGS sequencing, total content of cellular RNA was extracted from 80% confluence cells using an RNeasy Mini kit (Qiagen, USA) in accordance with the manufacture’s protocol. For cDNA synthesis, 1 μg of RNA was used as a template to reverse-transcribe the RNA into template DNA in accordance with the instructions of the ReverTra Ace qPCR RT kit (Toyobo, Japan). Polymerase chain reaction (PCR) was carried out to assess differences in mRNA expression of the aforementioned genes. The products were electrophoresed on a 2% agarose gel to visualize the differences in mRNA expression. Similarly, to further validate the results, a more sensitive quantitative PCR (qPCR) was carried out using SYBR green from Biorad.

### Transfection

To develop an overexpression vector for *MUC15*, the pMSCV puro (lentivirus) vector was used. *MUC15* gene was isolated from *MUC15*-expressing colon cancer cells and was used for complementary DNA (cDNA) synthesis. *MUC15* cDNA was cloned into pMSCV puro vector and transfected into Nthy-ori 3-1 cells, followed by puromycin treatment for selection.

Lentiviral constructs containing *MUC15*-specific shRNA conjugated with puromycin resistance genes and tGFP was obtained from Sigma Aldrich (MO, USA). To generate the lentivirus, tGFP-shRNA of Lenti vector and packaging vectors were used to co-transfect with HEK293T cells in accordance with the manufacturer’s instructions. Viral supernatants were harvested and used to transduce FTC-238 cells. Transduction efficiency was analyzed via qPCR and western blotting analyses.

### RNA sequencing

Total cellular RNA was extracted from cells grown to 80% confluence, using an RNeasy Mini Kit (Qiagen) in accordance with the manufacturer’s protocol. The total RNA was sent to Macrogen (Korea) for sequencing.

### Proliferation assay

Cells were seeded in triplicate wells in 96-well plates at 2000 cells per well and treated with PrestoBlue® Cell Viability Reagent (Invitrogen) for the proliferation assay in accordance with the manufacturer’s protocol. The experiment was performed every day for 7 days.

### Apoptosis assay

Cells were seeded in triplicate wells in six-well low-attachment plates at 5 × 10^4^ cells per well. After incubation for 1 days, 3 days, and 5 days, cells were harvested. Apoptosis was assayed using the Annexin-V apoptosis Detection Kit (eBioscience, UK) in accordance with the manufacturer’s instructions. Cells were washed once with 100 µl Binding Buffer and stained for 10 min with Annexin-V at room temperature in dark. Stained cells were washed once with 200 µl Binding Buffer and stained again with 7-Aminoactinomycin D. Stained cells were analyzed using a BD fluorescence-activated cell sorting Canto flow cytometer (BD Biosciences, UK).

### Sphere forming assay

Cells with greater stemness or self-renewal ability are likely to form spheres, which are more in number and bigger in diameter, when cultured in special media. This property was exploited in the following assay. Indicated cells were seeded at 5000 cells/well in triplicate in six-well low-attachment plates. Cells were cultured in Phenol-red free DMEM/F12 (Gibco) containing 1 ml of B27 supplement minus vitamin A (50×) (Gibco), 5 µl of rhEGF (100 µg/ml) (R&D System, UK), 100 µl of bFGF (10 µg/ml) (BD), 100 IU/ml penicillin, and 100 µg/ml streptomycin (Invitrogen). The cells were incubated for 5 days at 37 °C and 5% CO_2_ for sphere formation. UO126 was purchased from cell signaling technology (MA, USA). PTX and caffeine were purchased from Sigma Aldrich (MO, USA).

### Western blot analysis

Starvation was induced in the cells for 12 h in serum-free media. Thereafter, growth factor hEGF (100 ng/ml) (BD) was added and cells were incubated for 0, 10, 30, and 60 min. Thereafter, cells were lysed in RIPA lysis buffer containing 1 mL of RIPA buffer (10×) (Millionpore), 100 µl PMSF (Sigma), and 1 table of protease inhibitor. Collected the cell lysate were subject to spin-down by centrifuges at 13,000 × g for 15 min and only supernatant was used for western blot analysis. Protein lysates were quantified and mixed with 4X loading dye (10 ml of NuPAGE LDS Sample Buffer (4×) and 500 µl of beta-mercaptoethanol) at 20 µg/16 µl and heated at 95 °C for 5 min. Sixteen microliters of protein sample was loaded onto a polyacrylamide gel (12%), which was run at 100 V for 2 h and the proteins were then electroblotted onto Immuno-Blot PVDF Membrane (Biorad, USA) for 1 h at 250 mA, on ice. The membrane was then incubated with primary during overnight at 4 °C and secondary antibodies for 1 h at room temperature with agitation. ECL Prime Western blotting reagent (Amersham, UK) was used to develop the membrane. The primary antibodies were as follows: anti-*MUC15* (Novus, USA), anti-total *BRAF*, anti-phospho-*BRAF* (Ser445), anti-total *c-RAF*, anti-phospho-*c-RAF* (Ser338), anti-total *MEK*, anti-phospho-*MEK*, anti-total *ERK1/2*, and anti-phosphor-*ERK1/2* (Cell signaling technology, USA). β-actin was used as the control and detected with anti-β-actin rabbit polyclonal antibody (Sigma-Aldrich, USA). Anti-rabbit secondary antibody was purchased from Dako (USA).

### Statistical analysis

All quantified experimental data are expressed as mean ± SD values. To test statistical difference, student’s *t*-test and one-way ANOVA were conducted. GraphPad Prism 7.0 (GraphPad Software Inc., San Diego, CA, USA) was used for statistics analysis and *P < 0.05, **P < 0.01, and ***P < 0.001 values were used as significant values.

## Electronic supplementary material

Supplemental Figures

NGS data set for MUC15 vs. Nthy-ori-3-1 filtered by 2 fold
