# Supplementary material for: Promotion of tumor progression and cancer stemness by MUC15 in thyroid cancer via the GPCR/ERK and integrin-FAK signaling pathways
Source: Oncogenesis. 2018 Nov 12;7(11):85. doi: 10.1038/s41389-018-0094-y (PMC6232104; doi:10.1038/s41389-018-0094-y)
Supplement: Supplementary file 1 — Supplemental Figures [file 41389_2018_94_MOESM1_ESM.docx]

Promotion of tumor progression and cancer stemness by MUC15 in thyroid cancer via the GPCR/ERK and integrin-FAK signaling pathways

Cheolwon Choi^1†^, Tran Nguyen Thi Thao^1†^, Trinh Van Ngu^1†^, Sae Woong Park^2^, Min Suk Song^3^, Sung Hyun Kim^4^, Yun-Ui Bae^1^, Penchatr Diskul Na Ayudthaya^1^, Eunbit Kim^1^, Sujung Song^1^, Seongho Ryu^1^* and Kee-Hyun Nam^5^*

^1^Soonchunhyang Institute of Med-bioscience (SIMS), Sunchunhyang University, Chonan, Korea

^2^Department of Microbiology & Immunology, Weill Cornell Medical College, New York, USA

^3^Synaptic Circuit Plasticity Laboratory, Department of Structure & Function of Neural Network, K

orea Brain Research Institute, Daegu, Korea

^4^Department of Physiology, Kyung Hee University, School of Medicine, Seoul, Korea

^5^Department of Surgery, Yonsei University College of Medicine, Seoul, Korea

† These authors contributed equally to this work

*Correspondence should be addressed to

Kee-Hyun Nam , Department of Surgery, Yonsei University College of Medicine, 50 Yonseiro Seodaemungu, Seoul, 120-752, Korea

Tel: 82-2-2228-2100; E-mail: [KHNAM@yuhs.ac](mailto:KHNAM@yuhs.ac)

Fax: 82-2-313-8289

Seongho Ryu, Soonchunhyang Institute of Med-bio Sciences (SIMS), Chonan-Si, Korea, 336-745.

Phone: +82-41-530-4839; E-mail: [ryu@sch.ac.kr](mailto:ryu@sch.ac.kr)


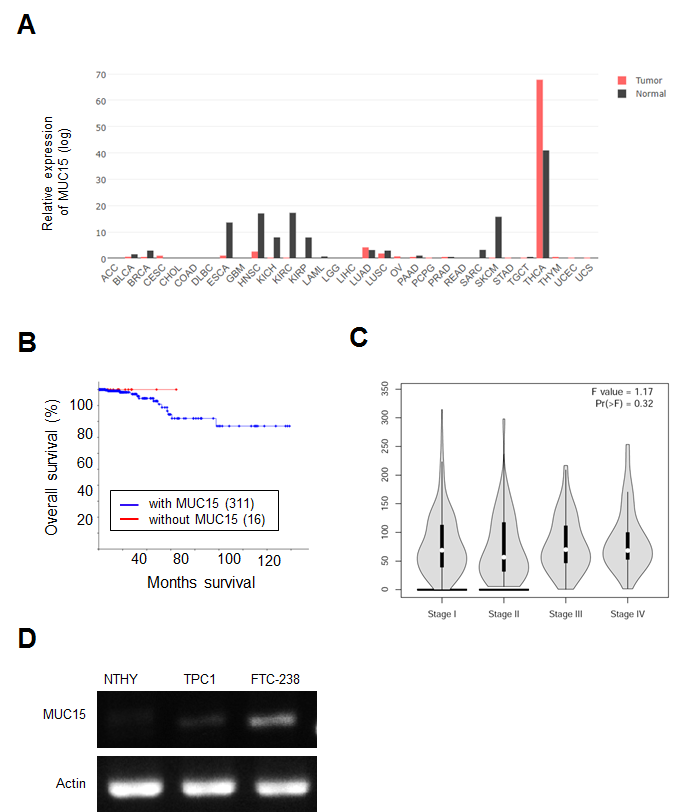


**Figure S1. Expression profiles of MUC15 in patient and thyroid cancer cells.** (A and C) MUC15 expression profiles from TCGA dataset of thyroid cancer patient using GEPIA website (http://gepia.cancer-pku.cn/)^1, 3^. THCA indicates TCGA project name of thyroid carcinoma (B) Patient with high MUC15 expression show worse prognosis analyzed by cBioPortal website (<http://www.cbioportal.org/>)^2^ (D) MUC15 expression at mRNA level among Nthy-ori-3-1, TPC-1 and FTC-238 cell lines


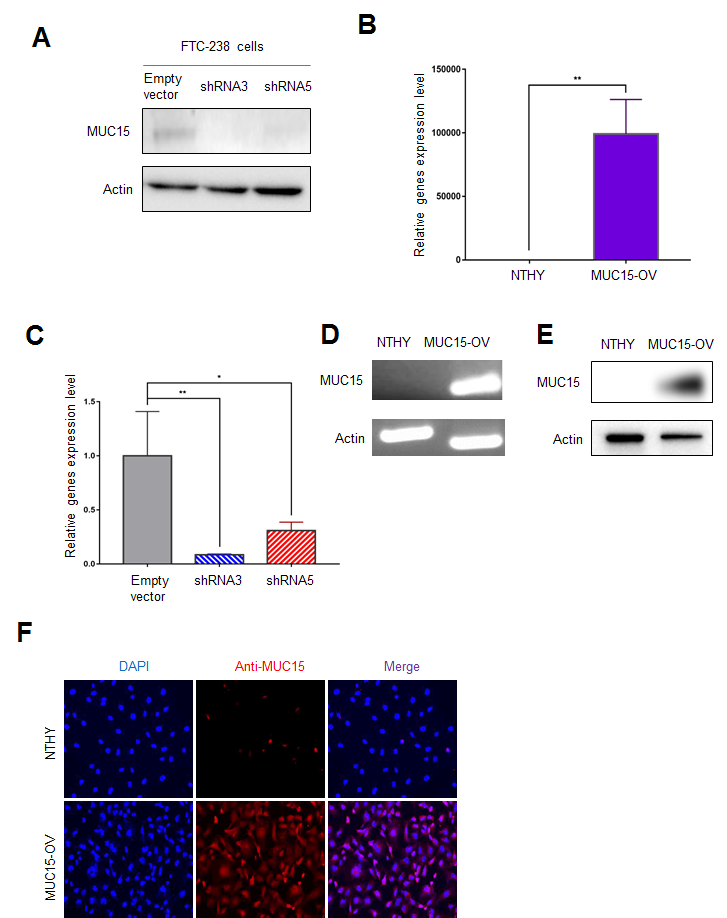


**Figure S2. Expression of MUC15 in knockdown cells and over-expressed cells.** (A) Immunobloting showing the expression of the MUC15 protein in the FTC-238 transfected with empty vector and two shRNA, named shRNA3 and shRNA5 respectively. (B). Quantitative expression level of MUC15 in FTC-238 cells and FTC-238 MUC15 knockdown cells. (C) Quantitative expression level of MUC15 in the Nthy-ori-3-1 and Nthy-ori-3-1 MUC15 overexpress cells. (D) RT-PCR analysis showing the higher expression of the MUC15 in the Nthy-ori-3-1 MUC15 overexpress cells comparing to Nthy-ori-3-1. (E) Immunostaining of MUC15 in in the Nthy-ori-3-1 and Nthy-ori-3-1 MUC15 overexpress cells. (F) The proliferation of Nthy-ori cell compared to MUC15 over expression cells in 2D and spheroid cell cultured condition.


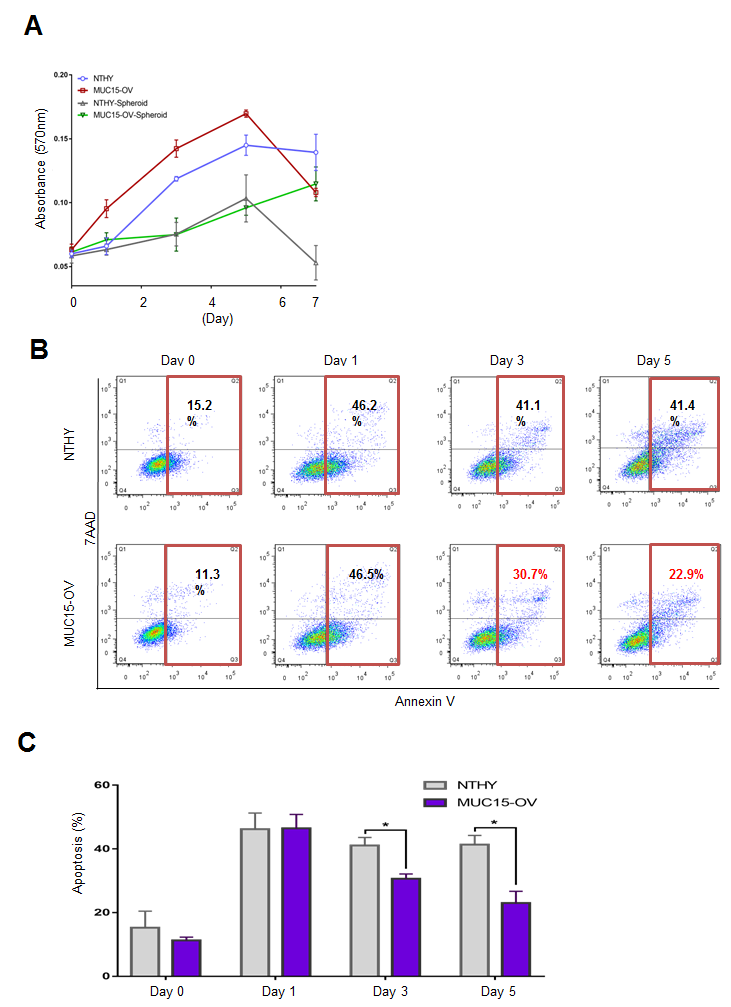


**Figure S3. Apoptotic of Nthy-ori cells compared to MUC15-ov cells.** (A) Measurement of proliferation between Nthy-ori-3.1 and MUC15-OV cells both attachment and detachment growth respectively. (B-C) Apoptosis rate of Nthy-ori-3-1 and MUC15-OV cells cultured in suspension condition in 5 days, analyzing by flowcytometry with 7AAD and Annexin-V conjugated with Pacific blue.


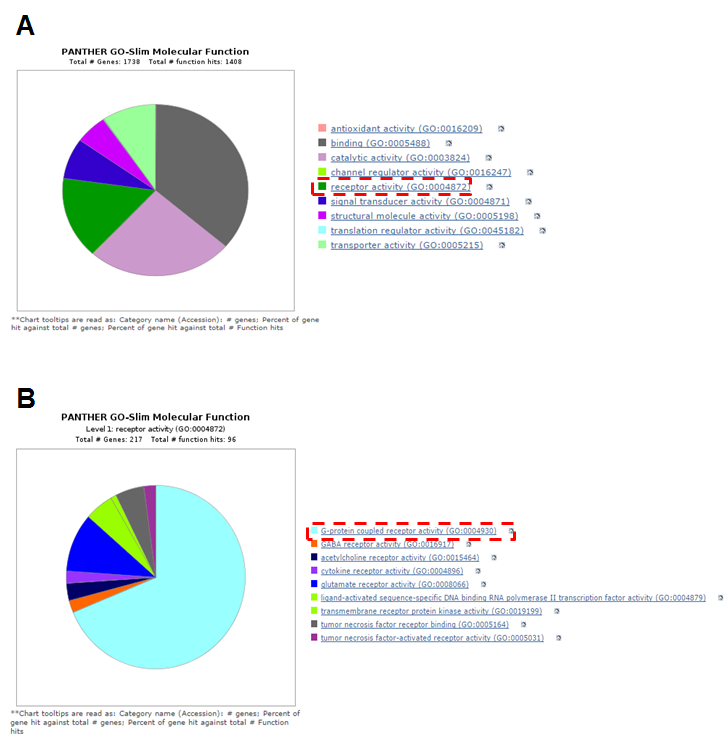


**Figure S4. Bioinformatic analysis with differentially expressed transcriptome profiles between Nthy-ori-3-1 and MUC15-OV cells**. (A-B) More than 4 fold increased genes in MUC15-ov cells comparing to Nthy-ori-3-1 were analyzed with Molecular function of GO term by PANTHER (<http://pantherdb.org/>) ^4^.


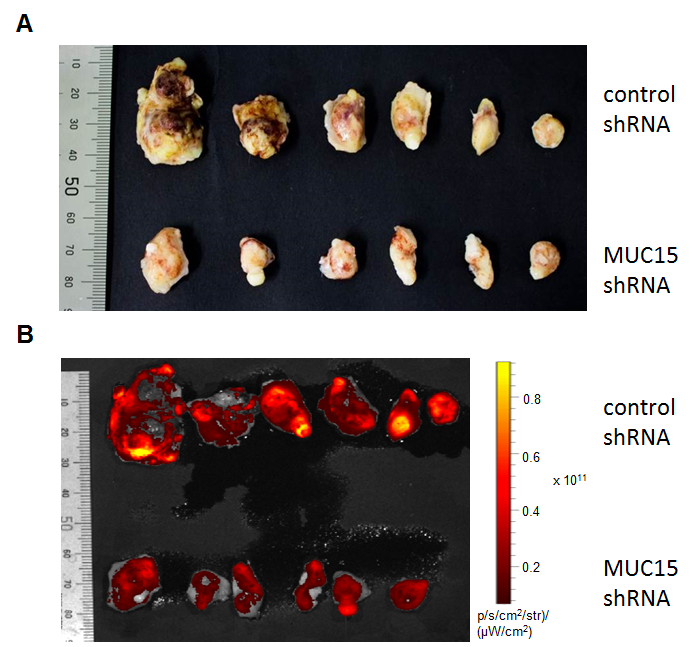


**Figure S5. *MUC15* expression enhanced tumorigenesis in NOD/SCID mice** (A) Actual size of tumor after injection thyroid cancer cells up to 30 days. **(**B) The GFP signal imaging after mouse were injected FTC-238 Control, FTC-238 *MUC15* knockdown (FTC-238-SH) cells u to 30 days respectively.


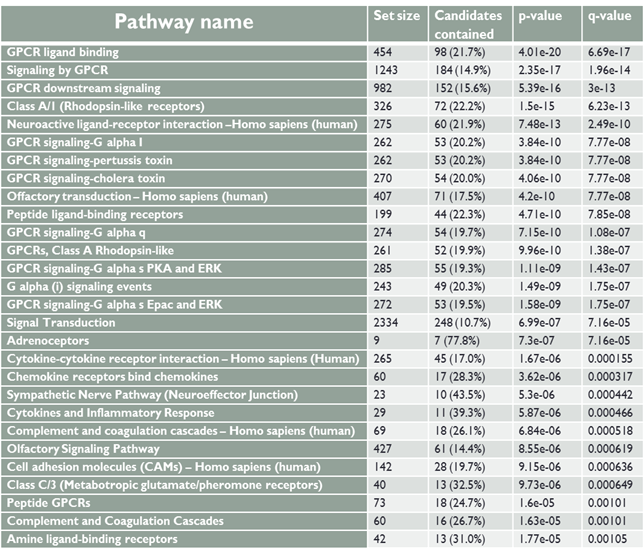


**Table S1. Genes that are highly expressed in MUC15 over-expression cells**. RNA-SEQ data was analyzed by CPDB.molgen website (<http://cpdb.molgen.mpg.de/>). Genes associated with G-protein-coupled receptor (GPCR) signaling pathway highly related to over-expression MUC15.

| GENE_SYMBOL | GENE NAME |
| --- | --- |
| HTR1A | 5-hydroxytryptamine receptor 1A(HTR1A) |
| HTR1B | 5-hydroxytryptamine receptor 1B(HTR1B) |
| HTR1F | 5-hydroxytryptamine receptor 1F(HTR1F) |
| CCL20 | C-C motif chemokine ligand 20(CCL20) |
| CCL27 | C-C motif chemokine ligand 27(CCL27) |
| CCR1 | C-C motif chemokine receptor 1(CCR1) |
| CCR5 | C-C motif chemokine receptor 5 (gene/pseudogene)(CCR5) |
| CCR7 | C-C motif chemokine receptor 7(CCR7) |
| CCR9 | C-C motif chemokine receptor 9(CCR9) |
| CXCL1 | C-X-C motif chemokine ligand 1(CXCL1) |
| CXCL13 | C-X-C motif chemokine ligand 13(CXCL13) |
| CXCL2 | C-X-C motif chemokine ligand 2(CXCL2) |
| CXCL3 | C-X-C motif chemokine ligand 3(CXCL3) |
| CXCR5 | C-X-C motif chemokine receptor 5(CXCR5) |
| ADCY8 | adenylate cyclase 8(ADCY8) |
| ADRA2A | adrenoceptor alpha 2A(ADRA2A) |
| ADRA2B | adrenoceptor alpha 2B(ADRA2B) |
| ADRA2C | adrenoceptor alpha 2C(ADRA2C) |
| APLNR | apelin receptor(APLNR) |
| BDKRB1 | bradykinin receptor B1(BDKRB1) |
| C3 | complement C3(C3) |
| C5AR1 | complement C5a receptor 1(C5AR1) |
| FPR2 | formyl peptide receptor 2(FPR2) |
| FPR3 | formyl peptide receptor 3(FPR3) |
| GRM3 | glutamate metabotropic receptor 3(GRM3) |
| GRM6 | glutamate metabotropic receptor 6(GRM6) |
| GRM8 | glutamate metabotropic receptor 8(GRM8) |
| HRH3 | histamine receptor H3(HRH3) |
| HCAR1 | hydroxycarboxylic acid receptor 1(HCAR1) |
| NMU | neuromedin U(NMU) |
| NPY | neuropeptide Y(NPY) |
| OPN1MW2 | opsin 1 (cone pigments), medium-wave-sensitive 2(OPN1MW2) |
| OXGR1 | oxoglutarate receptor 1(OXGR1) |
| PYY | peptide YY(PYY) |
| PTGER3 | prostaglandin E receptor 3(PTGER3) |
| P2RY4 | pyrimidinergic receptor P2Y4(P2RY4) |
| RGS7 | regulator of G-protein signaling 7(RGS7) |
| RXFP3 | relaxin/insulin like family peptide receptor 3(RXFP3) |
| SAA1 | serum amyloid A1(SAA1) |
| SSTR1 | somatostatin receptor 1(SSTR1) |
| SSTR2 | somatostatin receptor 2(SSTR2) |
| SSTR3 | somatostatin receptor 3(SSTR3) |
| S1PR4 | sphingosine-1-phosphate receptor 4(S1PR4) |
| TAS2R10 | taste 2 receptor member 10(TAS2R10) |
| TAS2R13 | taste 2 receptor member 13(TAS2R13) |
| TAS2R39 | taste 2 receptor member 39(TAS2R39) |
| TAS2R46 | taste 2 receptor member 46(TAS2R46) |
| TAS2R7 | taste 2 receptor member 7(TAS2R7) |
| TAS2R9 | taste 2 receptor member 9(TAS2R9) |

**Table S2. Genes that highly expressed in MUC15-ov cells related to GPCR pathway categorized with GO-term analysis.** Genes that distinctively expressed more than 2 fold in MUC15-ov cells were analyzed with GO-term ontology methods from RNA-SEQ results. Table contains list of target genes categorized in GPCR signaling pathway by GO-term analysis.

**Reference**

1 Agrawal N., *et al* (2014). Integrated genomic characterization of papillary thyroid carcinoma. *Cell* **159:** 676-690.

2 Gao J., *et al* (2013). Integrative analysis of complex cancer genomics and clinical profiles using the cBioPortal. *Sci Signal* **6:** pl1-pl1.

3 Tang Z, Li C, Kang B, Gao G, Li C, Zhang Z (2017). GEPIA: a web server for cancer and normal gene expression profiling and interactive analyses. *Nucleic acids research* **45:** W98-W102.

4 Thomas PD., *et al* (2003). PANTHER: a library of protein families and subfamilies indexed by function. *Genome research* **13:** 2129-2141.
